# Parental attachment and emotional intelligence mediates the effect of childhood maltreatment on callous-unemotional traits among incarcerated male adolescents

**DOI:** 10.1038/s41598-022-25285-0

**Published:** 2022-12-08

**Authors:** Jiaxi Peng, Huijie Lu, Jiaxi Zhang, Weizhuo Yuan, Peng Fang, Jianquan Tian, Lei Wang

**Affiliations:** 1 School of Psychology, Beijing Sport University, Beijing, China; 2 Department of Military Medical Psychology, Air Force Medical University, Xi’an, China; 3 Xi’an Research Institute of High-Technology, Xi’an, China; 4 Navel Medical Center of PLA, Shanghai, China; 5 Department of Medical Psychology, Strategic Support Force Medical Center, Beijing, China

**Keywords:** Psychology, Human behaviour

## Abstract

This study aimed to examine the impact of childhood maltreatment on callous-unemotional (CU) traits among incarcerated male adolescents, focusing primarily on the roles of parental attachment and emotional intelligence. A total of 454 male incarcerated adolescents from two juvenile correctional facilities, ranging in age from 14 to 18 years, completed a set of questionnaires consisting of a childhood trauma questionnaire, parent-attachment scale, emotional intelligence scale, and the Inventory of CU traits. The results revealed that childhood maltreatment, parental attachment, and emotional intelligence were all correlated with CU traits. Structural equation modeling analysis and the bootstrap test indicated that parental attachment and emotional intelligence mediated, in part, the effect of childhood maltreatment on CU traits. These findings expand the outcomes of previous research and shed light on how childhood maltreatment is related to CU traits.

## Introduction

Psychopathy, a type of personality disorder, is characterized by self-centeredness, cheating, impulsivity, and lack of empathy or guilt^1^. The callous-unemotional (CU) traits are the core affective features of psychopathy^2^. As a stable personality trait, CU traits primarily involve four features: lack of remorse or guilt, callousness and lack of empathy, disregard for performance, and shallow or deficient affect^2,3^. Previous studies have suggested that people with higher CU traits are more prone to criminal behaviors, especially violent crimes^4^. So far, the probable cause of CU traits has not been determined. On the one hand, the stability of CU traits suggest the possibility of being genetically determined. In support of this, Fontaine et al*.* followed 9462 twins and evaluated their CU traits at the ages of 7, 9, and 12^5^. Their findings suggest distinct developmental trajectories of CU traits from childhood to early adolescence, which are, in most cases, influenced by genetic factors^5^. On the other hand, environmental factors and life experience are also have a significant impact on CU traits. Stringent parenting style or lack of care in a home environment is associated with a high level of CU traits^6^, indicating that CU traits are affected by the environment. Kumsta and colleagues reported that high levels of CU traits typically existed in the absence of antisocial behavior in a group of children who have experienced early deprivation^7^. CU traits in these children may be caused by early formative experience rather than genetic vulnerabilities, as previously reported for community samples^8^.

In this study, we discuss the effect of environmental factors, particularly childhood maltreatment, on CU traits among incarcerated adolescents.

### Childhood maltreatment and callous-unemotional traits

Childhood maltreatment, which is a typical and persistent negative life event in the growth process of children, includes various forms of physical and emotional neglect, as well as physical, emotional, and sexual abuse^9,10^. Research indicates that childhood maltreatment has a close relationship with cognitive and emotional problems and is related to antisocial personality, aggressiveness, crime, conduct disorder, and substance abuse^11,12^. Childhood maltreatment is also closely correlated with CU traits scores in adulthood^13^. Dackis et al*.* found that CU traits are significantly correlated with childhood maltreatment, and that people who reported experiencing childhood maltreatment also reported higher CU traits^14^. Chang et al*.* found through the use of mediation analysis that childhood sexual abuse had effects on CU traits and violent delinquency in juvenile offenders, and that CU traits stemming from childhood sexual abuse significantly influenced violent delinquency in juveniles^9^. Using similar methods, Fang et al*.* documented that the presence of CU traits in victims of childhood maltreatment were a key predictor for the commission of adolescent cyberbullying^15^. Based on these reports, we hypothesize that *childhood maltreatment is significantly correlated with the CU traits of incarcerated adolescents, and those experiencing more childhood maltreatment may report higher CU traits* (Hypothesis 1)*.*

### The mediating role of parental attachment

Although previous studies have found significant correlation between childhood maltreatment and CU traits, there are insufficient data to determine how childhood maltreatment can predict CU traits and its inherent mechanism. Attachment theory holds that, before learning to talk, infants already possess developed internal working models (IWMs) during interactions with their caregivers, and such IWMs affect their personality development and behavioral patterns^16^. Experiencing childhood maltreatment damages the formation of intimate relationships, leading to insecure attachment^17^. Specifically, those who have had childhood maltreatment have low emotional expectation from others; they feel unworthy of being loved; and they are unable to develop positive relationships or satisfactory emotional experiences. The attachment relationship between parents and children plays a key role in a child’s development^18^. A secure and high-quality parental attachment can predict the positive development of children, such as excellent adaptation to campus life, confidence in interpersonal interactions, and pursuit of occupational goals^19^. In contrast, insecurity and low-quality parental attachment are significantly correlated with teenage depression, anxiety, suicidal behaviors, legal violation, drug abuse, and crime^18^.

The relationship between attachment and CU traits has been extensively discussed, and it is widely agreed that insecure attachment is significantly associated with CU traits^20^. Rehder et al*.* suggested that parental attachment was associated with empathic functioning, experiences of guilt, and conscience development, so attachment disorganization is a correlate of CU traits^20^. Pasalich and colleague found that, among children with behavioral problems, those with higher scores on CU traits appeared to be at increased risk of experiencing disruptions in parent–child attachment relationships^21^. A significant correlation between attachment and CU traits can also be found in the toddler period^22^.

In summary, childhood maltreatment leads to insecurity and low-quality parental attachment, which is significantly related to CU traits. Thus, we hypothesize that *parental attachment can mediate the effect of childhood maltreatment on CU traits* (Hypothesis 2)*.*

### Mediating effects of parental attachment and emotional intelligence

Emotional intelligence, which refers to the ability to perceive and handle emotions and to utilize emotional information to guide behaviors, was also found to have a close relationship with CU traits^23^. Those with higher emotional intelligence exhibit lower CU traits^24^. Sharp et al*.* also found that CU traits are associated with deficits in recognizing complex emotions^25^. Researchers found that children with high levels of CU traits had high levels of cognitive empathy and strong ability to understand others, but low levels of emotional empathy and low ability to share others’ emotions^25,26^. This indicates that high CU individuals understand other people’s emotions, but they do not share these emotions, leading to more callous reactions.

Childhood maltreatment and parental attachment can both significantly predict emotional intelligence. Previous studies report that individuals suffering more childhood maltreatment experience more difficulty in expressing their emotions as well as identifying and evaluating situations and other individuals^27,28^. Childhood maltreatment can decrease emotional intelligence; children experiencing maltreatment may be unwilling to express their feelings, and they may mistakenly consider the emotion expressed by other people as negative or a potential risk signal^28^. Zhao et al*.* also found that childhood maltreatment can significantly predict malicious envy and emotional intelligence^27^. Regarding attachment, people with secure attachment are more capable of expressing their emotions, managing and controlling their emotions, and can capably maintain interpersonal relationships^29–31^. Thus, we hypothesize that *insecure parental attachment and stunted emotional intelligence mediate the development of CU traits in individuals who suffer childhood maltreatment* (Hypothesis 3)*.*

Accordingly, we aimed to explore the effect of childhood maltreatment on the development of CU traits by surveying incarcerated adolescents, with a focus on parental attachment and emotional intelligence in the relationship between them.

## Methods

### Participants

In an urban province in China, 454 male incarcerated adolescents, ranging in age from 14 to 18 years (average age 16.38 years, standard deviation 1.39 years), from two juvenile correctional facilities participated in this study. Paper–pencil tests were conducted to collect data during their rest time in the reading room. In total, 454 copies of the questionnaire were distributed, and 429 valid copies were returned (25 copies were excluded because of an obvious pattern of answers, such as choosing A for all items). Among the valid responders, their types of offense included affray (21.1%), intentional injury (27.6%), forcible rape (17.3%), and robbery (34.0%). With regard to education, 97 (22.6%) participants completed primary school, 201 (46.9%) received junior high school education, and 131 had attended senior middle school or vocational high school (30.5%). There were 298 participants who reported rural household registration, accounting for 69.5% of the total.

The research described in this paper meets the ethical guidelines of Chengdu University and has been approved by its ethics committee (No. CDU210028). All methods were performed in accordance with the relevant guidelines and regulations. All participants had read and signed the informed consent form before participating in the study and were awarded 15 RMB (almost 2.5 USD).

### Instruments

#### The Childhood Trauma Questionnaire-Short Form (CTQ-SF)

The CTQ-SF, developed by Bernstein et al*.*^10^ and consisting of 28 items, was used to evaluate childhood maltreatment. Sample items are as follows: “People in my family said hurtful or insulting things to me” and “I was punished with a belt, a board, a cord, or some other hard object.” Responses were made using a 5-point scale, ranging from 1 = *never* to 5 = *always.* The CTQ-SF assesses the following five dimensions: physical neglect, physical abuse, emotional abuse, sexual abuse, and emotional neglect. The CTQ-SF, which was translated into Chinese, has been widely used in previous studies^32^. In the current study, Cronbach’s α coefficients of the five subscales were 0.73, 0.72, 0.74, 0.77, and 0.77, respectively.

#### Measurement of parental attachment

Parental attachment was evaluated by the paternal and maternal attachment subscales from the Inventory of Parent and Peer Attachment-Revised version, each consisting of 25 items^33^. Several examples are as follows: “My father encourages me to talk about my difficulties” and “When I am angry about something, my mother tries to be understanding.” The response were made using a 5-point scale, ranging from 1 = *Never* to 5 = *always*. After reverse scoring the negatively worded items, the score of each of the 25 respective items was averaged. Higher scores indicated higher quality of parental attachment. In the current study, Cronbach’s α coefficients of the paternal and maternal attachment subscales were 0.86 and 0.79, respectively. The scale, which was translated into Chinese, showed excellent validity and reliability^19^.

#### The Wong and Law Emotional Intelligence Scale (WLEIS)

The WLEIS, developed by Law et al*.*^34^, consisting of 16 items, was used to evaluate emotional intelligence. Sample items are as follows: “I am quite capable of controlling my own emotions” and “I always encourage myself to try my best.” The responses were made using a 7-point scale, ranging from 1 = *strongly disagree* to 7 = *strongly agree.* The WLEIS assesses the following four dimensions: self-emotion appraisal, other-emotion appraisal, use of emotion, and regulation of emotion. In the current study, Cronbach’s α coefficients of the four subscales ranged from 0.70 to 0.78.

#### Inventory of Callous-Unemotional Traits-Short Form (ICU-SF)

Hawes et al*.* selected 12 items using item response theory from the original Inventory of Callous-Unemotional Traits and, from them, developed the ICU-SF^35^. The validity and the reliability of ICU-SF have been supported in numerous subsequent studies of inmates^36^. The ICU-SF consists of two subscales measuring callousness and uncaring. Several example items are as follows: “I do not care about doing things well” and “I am concerned about others’ feelings.” Each item is rated using a 4-point scale, ranging from 1 = *Not at all true* to 7 = *Definitely true*. The scale was translated into Chinese, with one of the items “I hide my emotion from others” not suitable for measuring CU in the Chinese culture^37^. In the current study, Cronbach’s α coefficients of the two subscales were 0.79 and 0.77. The ICU-SF, which was translated into Chinese, showed excellent validity and reliability.

### Data analysis

Preliminary data analyses were conducted to examine the descriptive statistics (including means and standard deviations) and bivariate correlations. Then, a two-step procedure, introduced by Anderson and Gerbing^38^, was used to analyze the mediation effect. First, the measurement model of the four latent variables was tested to assess the goodness of fit represented by its respective indicators. Second, the maximum likelihood estimation was used to test the structural equation model (SEM). For the purpose of exploring the relationship among the four variables, a series of SEM analyses was conducted. We first tested the direct effect model in the relationship between childhood maltreatment on CU traits without mediators. Then, a partially mediated model with the two mediators of parental attachment and emotional intelligence in the relationship between them was examined. Finally, the bootstrap test was used to test the significance of the mediating effects. As recommended by Tofighi and Kelley^39^, the bootstrapping approach was adopted to examine the significance of indirect effects with data that are not normally distributed.

The following indices were adopted to measure the goodness of fit of models: (1) Chi-squared statistic (χ^2^), χ^2^/df; (2) standardized root mean squared residual (SRMR); (3) root mean square error of approximation (RMSEA); and (4) comparative fit index (CFI). Generally, χ^2^/df < 5, CFI > 0.95, SRMR < 0.08, and RMSEA < 0.08 indicate that the model has high goodness-of-fit at a significance level of p < 0.05^40^.

## Results

### Descriptive statistics and correlation analysis

The mean values, standard deviations, and intercorrelations for all observed variables are presented in Table 1.

**Table 1:** Means, standard deviations, and correlations of the observed variables (n = 429).

	Mean	SD	2	3	4	5	6	7	8	9	10	11	12	13
1. Callousness	1.98	0.53	0.38**	0.37**	0.37**	0.47**	0.38**	0.39**	− 0.23**	− 0.36**	− 0.26**	− 0.22**	− 0.24**	− 0.33**
2. Uncaring	2.13	0.58		0.28**	0.27**	0.34**	0.31**	0.31**	− 0.25**	− 0.26**	− 0.20**	− 0.17**	− 0.14**	− 0.23**
3.Physical neglect	1.80	0.75			0.66**	0.73**	0.78**	0.68**	− 0.33**	− 0.30**	− 0.16**	− 0.18**	− 0.16**	− 0.21**
4. Physical abuse	2.02	0.81				0.75**	0.73**	0.73**	− 0.25**	− 0.30**	− 0.18**	− 0.19**	− 0.19**	− 0.24**
5. Emotional abuse	1.94	0.76					0.80**	0.78**	− 0.31**	− 0.29**	− 0.28**	− 0.21**	− 0.25**	− 0.28**
6. Emotional neglect	1.78	0.74						0.78**	− 0.30**	− 0.29**	− 0.23**	− 0.20**	− 0.22**	− 0.26**
7. Sexual abuse	1.88	0.79							− 0.24**	− 0.26**	− 0.16**	− 0.15**	− 0.19**	− 0.21**
8. Paternal attachment	3.58	0.67								0.57**	0.19**	0.23**	0.18**	0.20**
9. Maternal attachment	3.62	0.61									0.21**	0.19**	0.17**	0.22**
10. Self-emotion	3.19	0.89										0.48**	0.47**	0.53**
11. Other-emotion	3.74	0.81											0.58**	0.51**
12. Use of emotion	3.59	1.00												0.67**
13. Regulation	3.42	0.96												

**p < 0.01.

### Measurement model

Confirmatory factor analysis was adopted to assess whether the measurement model fit the sample data adequately. The full measurement model included four latent constructs (childhood maltreatment, parental attachment, emotional intelligence, and CU traits) and 13 observed variables. The measurement model fit the data very well: *χ*^2^/df = 1.92, p < 0.01; RMSEA = 0.05; SRMR = 0.03; and CFI = 0.98. All of the factor loadings for the indicators on the latent variables were significant (p < 0.01), indicating that all of the latent constructs were well represented by their indicators.

### Structural model

**Figure 1:**
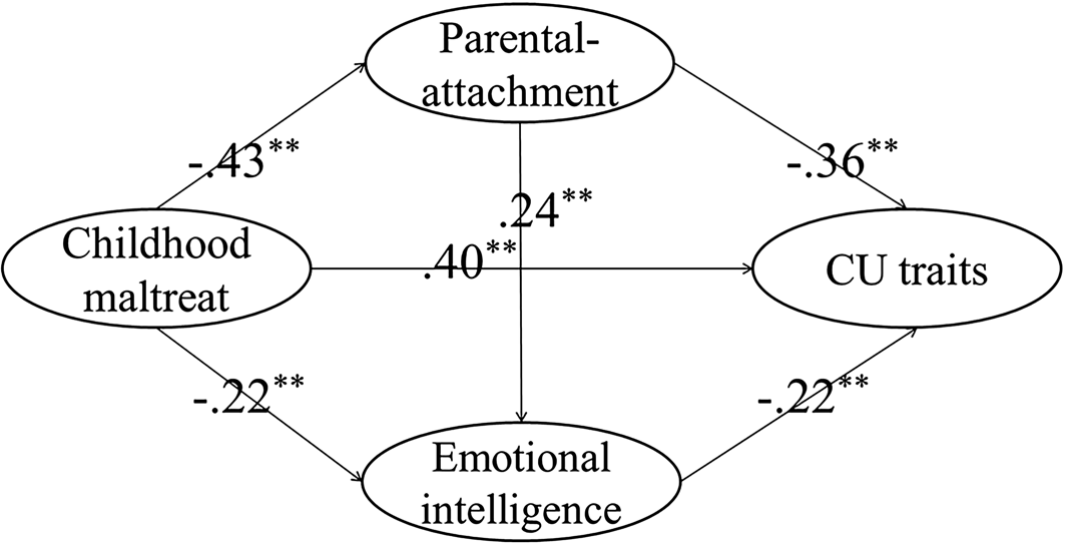
The final structural model.

The SEM was used to analyze the mediation effect. First, the direct effect of childhood maltreatment on CU traits without mediators was tested. The directly standardized path (β = 0.65, p < 0.01) was significant. Thus, Hypothesis 1 was supported in that there was a significant correlation between childhood maltreatment and CU traits. Then, a partially mediated model containing the mediators of parental attachment and emotional intelligence with a direct path from childhood maltreatment to CU traits, as well as a path from parental attachment to emotional intelligence, was tested (Fig. 1). The results showed that the model fit the data well (Table 2) and that all paths were significant. Thus, Hypothesis 2 was supported, i.e. parental attachment and emotional intelligence mediated the effect of childhood maltreatment on CU traits.

**Table 2:** Fitness indexes.

Model	*df*	*χ*^*2*^	*χ*^*2*^*/df*	CFI	RMSEA	SRMR
Model A	24	56.42	2.35	0.98	0.06	0.03
Model B	58	102.26	1.76	0.99	0.04	0.03

### The confidence interval of direct and indirect effects

The mediating effects of parental attachment and emotional intelligence between childhood maltreatment and CU traits were tested for significance using the bootstrap estimation procedure in AMOS (a bootstrap sample of 1000 was specified). Table 3 shows the direct and indirect effects and their associated 95% confidence intervals. As shown in Table 3, the indirect effect of childhood maltreatment on CU traits through parental attachment and emotional intelligence was significant, and the indirect effects accounted for 38.46% of the total. Thus, Hypothesis 3 was supported.

**Table 3:** Direct and indirect effects and 95% confidence intervals for the Model B (n = 429).

Model pathways	Estimated effect	95% CI	
		Lower bonds	Up bonds
**Total effect**			
Childhood maltreat → CU traits	0.65*	0.50	0.75
**Direct effect**			
Childhood maltreat → CU traits	0.40*	0.23	0.57
Childhood maltreat → parental-attachment	− 0.43*	− 0.30	− 0.54
Childhood maltreat → emotional intelligence	− 0.22*	− 0.07	− 0.37
Parental-attachment → emotional intelligence	0.24*	0.12	0.38
Parental-attachment → CU traits	− 0.36 *	− 0.20	− 0.55
Emotional intelligence → CU traits	− 0.22*	− 0.08	− 0.36
**Indirect effect**			
Childhood maltreat → (parental-attachment) → emotional intelligence	− 0.10*	− 0.04	− 0.18
Parental-attachment → (emotional intelligence) → CU traits	− 0.05*	− 0.02	− 0.10
Childhood maltreat → (parental-attachment, emotional intelligence) → CU traits	0.25*	0.13	0.35

*Empirical 95% confidence interval does not overlap with zero.

## Discussion

In the present study targeting incarcerated male adolescents, the effects of childhood maltreatment on CU traits were studied. The results show that parental attachment and emotional intelligence can, in part, mediate the effect of childhood maltreatment on CU traits. This study expands the outcomes in the previous research to some extent, especially with respect to how childhood maltreatment is correlated with CU traits.

Consistent with recent research, we find that childhood maltreatment is significantly correlated with two dimensions of CU traits^13,15^. The formation of CU traits is associated with biological factors (e.g., genetics and cerebral injury) and with psychological and social factors^5^. The formation of CU traits begins in childhood^41^, and early childhood traumas are related to many personality disorders^42^. As a persistent negative life event, childhood maltreatment certainly is significantly correlated to CU traits. According to Bandura’s social-learning theory, during the growth or socialization process, children will imitate the maltreatment imposed on them^43^. Individuals under long-term maltreatment and negligence tend to have intense negative emotions, low self-esteem^44^, and suffer isolation, depression, self-humiliation, and fury, which further leads to apathy, a deficit of sympathy, and the development of CU traits^8^.

This study demonstrates that both parental attachment and emotional intelligence can, in part, mediate the effect of childhood maltreatment on CU traits. Attachment theory holds that relationships and interactions with intimate others develop into the IWMs that will affect personality development and behavioral patterns^16^. Such IWMs determine whether the individuals feel they have value, whether they deserve attention and love from others, and whether other people are trustworthy^45^. Parental attachment is a major type of attachment, while childhood maltreatment is largely imposed on children by parents or other family members. Childhood maltreatment not only destroys parent*–*child relationships but also prevents the formation of high-quality parental attachment^46^. Negative parent*–*child relationships during early childhood predispose the affected individuals to have negative feelings toward others and the world, to feel that other people are unreliable and unsafe, and that they themselves are unloved^46^. Hence, they may suffer problems, including inhospitality, delayed moral development, aggressiveness, and antisocial behaviors—in short, CU traits^20^.

This study delineates the progression from childhood maltreatment, which engenders insecure parental attachment, which leads to stunted emotional intelligence, which in turn gives rise to CU traits. The IWMs of attachment people form from their parental relationships decide how people handle social stimulation and impact their emotional regulation, cognition, and interpersonal relationships^45^. Parental attachment affects emotional intelligence, which consists of evaluating, expressing, and regulating emotion^29–31^. Persons with CU traits have low emotional intelligence, displaying little empathy or sympathy for others, as well as poor control over their own emotions. Hence, childhood maltreatment, through the mediating effects of parental attachment and emotional intelligence, is related to CU traits.

As childhood maltreatment is an important contributing factor to CU traits, which are strongly associated with antisocial behavior, society and government should do everything possible to prevent child maltreatment in order to reduce juvenile crime—to say nothing of the lifelong mental, emotional, physical, and societal well-being of the children themselves. The occurrence and consequences of child maltreatment are considered to occur from a combination of individual, familial, and social factors^8,47^. For those who have experienced or who continue to experiencing maltreatment during childhood, focus should be placed on improving the relationships these children experience, providing them with positive relationships and parental care^19^. Parents of these children need to convey more security, and provide intimate and reliable signals to them, thereby promoting the formation of high-quality and secure attachment relationships with them^48^. Studies have found that the CU traits are more likely to decrease during childhood than adolescence, which indicates that childhood is a developmental stage more amenable to intervention and prevention efforts^49^. Thus, prevention programs specifically for young ages in schools that aim to train the emotion-controlling abilities and positive emotional regulation strategies for these childhood maltreatment populations will be more likely to lower the risks of CU traits in the subsequent growth period.

This study has several limitations. First, this study targets incarcerated male adolescents. This group was selected because of its high incidence of CU traits. Our findings are invaluable for the prevention of juvenile delinquency, but future studies are warranted to determine whether our findings can be expanded to the general juvenile population. Second, the fact that all questionnaires were completed by the same participants may cause common method variance. Third, we find that emotional intelligence and parental attachment can only partially explain the correlation between childhood maltreatment and CU traits and behaviors, indicating that there may be other mediating variables that should be further studied and researched.

In conclusion, the current study finds that childhood maltreatment significantly predicts CU traits, and that the effect of childhood maltreatment on CU traits is, in part, mediated by parental attachment and emotional intelligence. These findings may offer some clues that can motivate us to intervene with regard to the CU traits of teenagers in order to prevent them from committing crime.

## Data Availability

The datasets used and/or analyzed during the current study are available from the corresponding author on reasonable request.
